# Effectiveness of Integrated HIV Prevention Interventions among Chinese Men Who Have Sex with Men: Evaluation of a 16-City Public Health Program

**DOI:** 10.1371/journal.pone.0050873

**Published:** 2012-12-31

**Authors:** Shaodong Ye, Yan Xiao, Canrui Jin, Holly Cassell, Meridith Blevins, Jiangping Sun, Sten H. Vermund, Han-Zhu Qian

**Affiliations:** 1 Vanderbilt Institute for Global Health, Vanderbilt University, Nashville, Tennessee, United States of America; 2 National Center for AIDS/STD Control and Prevention, China Center for Disease Control and Prevention, Beijing, China; 3 Department of Biostatistics, Vanderbilt University, Nashville, Tennessee, United States of America; University of Alabama at Birmingham, United States of America

## Abstract

To examine the impacts of a multi-city HIV prevention public health program (China Global Fund Round 5 Project) on condom use and HIV infection, we analyzed four yearly cross-sectional surveys from 2006 through 2009 among 20,843 men who have sex with men (MSM) in 16 Chinese cities. Self-reported condom use at last sex with a male partner increased from 58% in 2006 to 81% in 2009 (trend test, *P*<0.001). HIV prevalence increased from 2.3% in 2006 to 5.3% in 2009 (*P*<0.001). Multivariable logistic regression analysis showed that self-reported receipt of interventions was an independent predictor of increased condom use at last sex with a male partner over time (adjusted odds ratio [aOR], 1.63 in 2006 to 2.33 in 2009; *P*<0.001), and lower HIV prevalence (aOR, 1.08 in 2006 to 0.45 in 2009; *P*<0.001). HIV prevalence increased from 2006–2009 for participants with no self-reported receipt of interventions (2.1% in 2006 to 10.3% in 2009) and less so for those with interventions (2.4% to 4.7%). This Chinese public health program had positive impacts on both behaviors and disease rate among MSM population. Escalation of the coverage and intensity of effective interventions is needed for further increasing condom use and for reversing the rising trend of HIV epidemic.

## Introduction

Men who have sex with men (MSM) comprise a large proportion of new HIV infections in Western countries. U.S. Centers for Disease Control and Prevention (CDC) reported that more than 60% of the newly diagnosed HIV cases in the United States in 2009 were MSM [1]. In the WHO European Region, the proportion of MSM among new HIV infections was estimated to be 20% in 2010 [2]. The epidemic has also increased among MSM populations in Africa and Asia; the UNAIDS report in 2010 showed that the HIV prevalence among MSM aged 15–49 years varied from 12% to 44% in seven countries in sub-Saharan Africa [3]. Though MSM are not the principal subgroup driving HIV transmission in Southeast Asia, the proportion of new HIV infections among MSM has also increased in several Southeast Asian countries [4]. In China, the proportion of MSM among new HIV infections rose from 12.2% in 2007 to 29.4% in 2011 [5], [6]. The increasing HIV epidemic among Chinese MSM has been confirmed by recent meta-analyses: the national average prevalence from cross-sectional studies was 5.3% (95% CI: 4.8%–5.8%) in 2009 [7], and average incidence rate from cohort studies was 3.5% (95% CI: 1.7%–5.3%) [8]. This changing epidemic among Chinese MSM is contributable to increasing social tolerance to homosexuality and growing social networks of MSM, high efficiency of HIV transmission through unprotected receptive anal sex, and stigma and discrimination as barriers to HIV care [9], [10].This serious trend suggests the need for urgent and effective interventions among MSM globally, and in China in particular given the size and mobility of its population.

In response to the HIV/AIDS epidemic, the Chinese government has passed new laws and regulations, invested funds, and trained personnel for disease prevention and control, particularly since 2003 when the SARS outbreak exposed the weakness of the Chinese public health system [11]. Notable was the Regulation on the Prevention and Treatment of HIV/AIDS, enacted by The State Council in 2006, that outlined the legal obligation of the government to make available comprehensive HIV/AIDS prevention intervention and treatment programs [12]. Subsequently, China has invested substantial funds and human resources into public health programs for HIV/AIDS prevention and treatment, such as needle exchange and methadone maintenance programs for injection drug users [13], [14], and free combination antiretroviral therapy (cART) programs for rural and/or poor urban residents living with HIV/AIDS [15], [16]. China has also used technical support and funds from international organizations and other developed countries, especially the Global Fund to Fight AIDS, Tuberculosis and Malaria (GF). By 2011, China had successfully obtained the Global Fund round 3, 4, 5, 6, and 8 projects (China GF-3, 4, 5, 6 & 8), and a combined one (China Consolidated GF Program) that aimed to scale up HIV/AIDS prevention, treatment and care in China to achieve universal access for high risk populations and PLWHA.

The China GF-3 program, popularly known as the China Comprehensive Aids Response (China CARES), provided HIV treatment, care and prevention service to infected plasma donors and their families in 7 provinces in central China [17], [18]. China GF-4 aimed to reduce HIV transmission among injection drug users (IDUs) and commercial sex workers (CSWs) in 7 high prevalence provinces. China GF-5 focused on preventing a new wave of the HIV epidemic among MSM and other high risk groups in six low-prevalence provinces and one municipality (Chongqing), and is the source of data for this study. China GF-6 was designed for mobilizing civil society to scale up HIV/AIDS control efforts in key vulnerable populations across the country. China GF-8 aimed to reach vulnerable migrants with HIV/AIDS prevention and care services in seven provinces.

Public health projects, like those of the China GF investments, usually focus on delivering interventions and services. These projects may collect a large volume of process and outcome data, much of which is never fully analyzed and published. To better assess the full impact of the China GF-5 program, we studied condom use and HIV and syphilis prevalence among MSM over four project years (2006 to 2009).

## Methods

### Data source

We analyzed yearly cross-sectional surveys among MSM performed on behalf of the China GF-5 project. All analyses used fully de-identified data and our protocol was reviewed and approved by the institutional review boards of the National Center for STD/AIDS Prevention and Control of China CDC and Vanderbilt University.

### China GF-5 project

**Figure 1:**
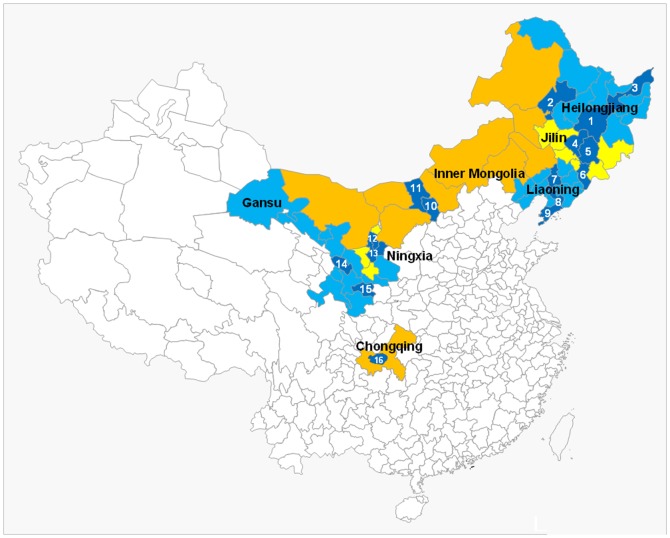
China GF-5 project sites: Location of the 15 cities in 6 provinces and Chongqing Municipality in China. **Each number represents a study city.**

The China GF-5 project sought to prevent a new wave of HIV in 6 low-prevalence provinces (Heilongjiang, Jilin, Liaoning, Inner Mongolia, Ningxia, Gansu) and one municipality in China (Chongqing) (Figure 1). Sixteen cities were included with 2–3 cities from each province and three districts from Chongqing Municipality. Each province included one capital city and one or two prefectural-level cities. Prefectural-level cities were selected based on cooperation of local health departments and experience of access to MSM population. The target populations included the four HIV aforementioned populations: MSM, CSWs, IDUs and migrants, and interventions were implemented during 2006 and 2009.

#### HIV prevention interventions

The intervention activities in China GF-5 included: (1) policy work to nurture supportive social and policy environments for HIV intervention and treatment; (2) implementation of multiple intervention approaches, including peer education, outreach interventions, provision of free condoms, and free voluntary HIV counseling and testing; (3) provision of enhanced sexually transmitted infection (STI) services and management for the four vulnerable populations, including care referral services for HIV-positive people; (4) capacity-building for community-based organizations (CBO); and (5) enhancement of behavioral surveillance, monitoring and evaluation.

The intervention activities targeted MSM who were living in the project cities included distribution of health information and provision of free condoms, free HIV testing and counseling, STI referral service, and antiretroviral (ARV) treatment. Intervention was delivered by peers, public health staff and clinicians through multiple approaches, including clinic-based, community outreach, instant message, Internet-based (website, chat room).

#### Project evaluation

Data for the project evaluation were collected in 4 rounds of annual cross-sectional epidemiological surveys: July–September, 2006; July–September, 2007; April–June, 2008, and April–June, 2009. Men aged 18 years or older with self-reported anal or oral sexual behavior with a male partner in the past 12 months and willingness to provide informed consent and take HIV testing were eligible.

Most participants in these surveys were recruited using a snowball sampling method [19]. Respondent-driven sampling [20] was used in the 2008 and 2009 surveys in all three Chongqing districts and in Changchun and Harbin cities. Based on interviews with key informants and geographic mapping, the local project teams in each city compiled a list of places that MSM frequented, including bars, public bathrooms, parks, and public Internet rooms. An attempt was made to recruit a sample of participants that was proportional to the estimated number of MSM in each venue. About 5,000 MSM were recruited in each survey (n = 20,843 for all four rounds) (Table 1).

**Table 1:** Number of men who have sex with men (MSM) who participated in yearly surveys in 16 Chinese cities from 2006 to 2009.

City	Year				Total
	2006	2007	2008	2009	
Anshan	294	402	405	402	1,503
Baotou	57	253	406	367	1,083
Changchun	397	500	492*	450*	1,839
Dalian	350	415	401	403	1,569
Harbin	390	419	451*	450*	1,710
Huhehaote	130	146	300	304	880
Jinlin	397	405	300	300	1,402
Jiamusi	407	297	302	300	1,306
Lanzhou	264	130	200	200	794
Qiqihar	398	402	300	300	1,400
Shenyang	395	407	450	450	1,702
Tianshui	174	206	200	200	780
Tonghua	399	259	304	400	1,362
Wuzhong	17	0	35	0	52
Yinchuan	55	0	312	200	567
Chongqing	952	740	602*	600*	2,894
Total	5,076	4,981	5,460	5,326	20,843

* Participants were recruited using respondent driven sampling.

#### Data collection

Each participant completed a questionnaire interview on demographics, HIV knowledge and attitudes, sexual behaviors, history of STIs, history of HIV testing, and prior experience of receiving HIV/STI counseling and intervention services. Venous blood specimens were collected anonymously from all participants for HIV and syphilis testing. An enzyme-linked immunosorbent assay (Vironostika HIV Uni-Form II Ag/Ab; BioMérieux Corporate, Marcy l'Etoile, France) was used for screening HIV antibody in the 2006 and 2007 surveys and a rapid test (HIV (1+2) Antibody (Colloidal Gold), KHB Shanghai Kehua Bio-engineering Co, Ltd) was used in the 2008 and 2009 surveys. Positive specimens were confirmed for HIV infection using Western blot (HIV Blot 2.2 WB; Genelabs Diagnostics, Singapore). Rapid Plasma Reagent (Beijing Wantai Biologic Production Company, Beijing, China) rapid testing was used for syphilis screening test, and the *Treponema Pallidum* Particle Agglutination Assay (Treponema pallidum Antibodies; Rong Sheng Biostix Inc, Shanghai, China) was used for confirmatory testing [21], [22]. The participants with syphilis infection were referred to local STI clinics, and those with HIV infection received post-test counseling and were assessed for cART eligibility and were recommended for enrollment in free cART programs in local CDCs.

### Data analysis

#### Study hypotheses and sample size

The null hypotheses in our analyses were: *hypothesis 1*: there was no difference of condom use at last sex with a male sexual partner between post-intervention survey in 2009 and baseline survey in 2006; and *hypothesis 2*: there was no difference of HIV prevalence between post-intervention and baseline surveys. We anticipated that interventions would increase condom use, and if this behavioral change efficiently translated into disease rate, new infections of HIV would significantly reduce and therefore HIV prevalence in MSM population would remain stable over the project years.

#### Analysis methods

The distributions of categorical variables across 4 rounds of surveys were compared using Pearson's chi-square test. Median and interquartile range [IQR] were calculated for continuous variables, and their distributions were compared using Kruskal-Wallis test. The Cochrane-Armitage trend test was used to examine the trend of condom use or HIV and syphilis prevalence rates over 4 project years; these tests are valid with large sample sizes in each survey.

As the China GF-5 project did not have a formal comparison group, we were interested in whether the changes in condom use at last sex or HIV prevalence over four project years were associated with exposure to any of the six project interventions, which included provision of free condoms, provision of free lubricants, peer education, STI testing and treatment, HIV testing and counseling, and distribution of AIDS/STI publicity materials (pamphlets, brochure, booklet, or foldout). The coverage rate was calculated based on receipt of any of these six interventions; meanwhile, a composite intervention score was created with one point for receiving each intervention in the past 12 months, therefore the score had a value ranging from 0 to 6. We used multivariable logistic regression to determine characteristics of participants associated with any receipt of intervention.

Both univariable and multivariable logistic regression analyses were performed pooling all participants in the four surveys, examining what factors were independently associated with condom use or with HIV infection. In addition to self-reported receipt of any intervention, predictors included in the models were study year, age, marriage, education, ethnicity, residence, occupation, recruitment venue, age at first sex with a man, and 6-month drug use. Initially, 6-month frequency of male sex partners, STI prevalence, and HIV knowledge were included; however, these variables are also targets of intervention efforts and their modifying effects on condom use or HIV prevalence are not of interest, thus they were dropped from inclusion. An interaction term between study year and intervention receipt was included in both models. Robust variance estimates were used in all models, as the respondents may be correlated (ie, men who may have been interviewed in more than one survey and correlation within community). All analyses were performed using the software of Stata® version 11 (Stata Corp LP, College Station, TX, USA) and SAS® 9.3 (SAS Institute Inc., Cary, NC, USA).

## Results

### Demographics of MSM participants

The age of participants ranged from 18 to 77 years, with a median of 27 or 26 years in all four surveys. The median age of first sex with a man was 23 or 24 years, about 4 years older than that of first sex with any sexual partner (Table 2). While a quarter (26%) of men were married in the 2006 survey, this was only 18% in the 2009 survey; about one third self-identified as bisexuals. About 92% were of Han ethnicity, a rate consistent with the general population in China. About one third of participants had received college education, and 15%–17% in each survey were currently enrolled in colleges. Local residents accounted for 70% of respondents. Nearly two-thirds (65%) of MSM had multiple sexual partners, and <2% reported using illicit drugs. The self-reported rate of STI symptoms and signs (e.g., painful urination, cloudy or bloody discharge from the penis, and ulcers and warts on the penis and around anus) decreased by half from 18% in the 2006 survey to 9% in 2009. The socio-demographics of participants were compared between those recruited via RDS in the 2008 and 2009 surveys and those recruited via snowball sample in the 2006–2007 survey in Chongqing, Changchun, and Harbin cities, and no significant difference was observed (not shown in Table 2).

**Table 2:** Demographics and sexual behaviors of MSM surveyed in 16 Chinese cities from 2006 to 2009.

Variable	2006 (n = 5076, %)	2007 (n = 4981, %)	2008 (n = 5460, %)	2009 (n = 5326, %)
Age, years* (median, IQR)	27 (23–34)	26 (22–34)	26 (22–34)	26 (22–35)
Age of first sex, years (median, IQR)*	19 (17–21)	19 (17–21)	20 (18–22)	20 (18–22)
Age of first sex with a man, years (median, IQR)*	23 (20–27)	23 (20–27)	23 (20–28)	24 (20–30)
Marital status				
Married	1,324 (26.1)	1,192 (23.9)	1,122 (20.5)	986 (18.5)
Cohabitating	121 (2.4)	34 (0.7)	110 (2.0)	45 (0.8)
Single	3,269 (64.4)	3,527 (70.8)	3,897 (71.4)	3,897 (73.9)
Divorced	361 (7.1)	227 (4.6)	331 (6.1)	361 (6.8)
Ethnicity				
Han	4,684 (92.3)	4,560 (91.5)	5,005 (91.7)	4,915 (92.3)
Other	392 (7.7)	421 (8.5)	455 (8.3)	411 (7.7)
Education				
Middle school or below	1,188 (23.4)	1,212 (24.3)	1,375 (25.2)	1,580 (29.7)
High school	1,926 (37.9)	2,022 (40.6)	2,169 (39.7)	2,049 (38.5)
Some college or higher	1,962 (38.7)	1,747 (35.1)	1,916 (35.1)	1,697 (31.8)
Local resident**	1,377 (27.1)	1,147 (23.0)	1,867 (34.2)	1,734 (32.6)
Self-reported sexual orientation				
Homosexual	3,089 (60.9)	3,077 (61.8)	3,030 (55.5)	3,498 (65.7)
Heterosexual	74 (1.4)	81 (1.6)	78 (1.4)	53 (1.0)
Bisexual	1,599 (31.5)	1,607 (32.3)	2,106 (38.6)	1,675 (31.4)
Unknown	314 (6.2)	216 (4.3)	246 (4.5)	100 (1.9)
Recruitment venue				
Bars	1,853 (38.1)	1,292 (25.9)	1,380 (25.3)	1,228 (23.1)
Saunas	882 (18.2)	915 (18.4)	955 (17.5)	937 (17.6)
Parks	502 (10.3)	729 (14.6)	693 (12.7)	831 (15.6)
Internet or other	1,620 (33.4)	2,045 (41.1)	2,426 (44.5)	2,321 (43.7)
Number of male sex partners in the past 6 months				
0 or 1	1,231 (34.2)	1,156 (30.8)	1,587 (36.2)	1,685 (36.8)
≥2	2,366 (65.8)	2,602 (69.2)	2,797 (63.8)	2,893 (63.2)
Occupation				
Students	898 (17.8)	833 (16.7)	837 (15.3)	795 (14.9)
Non-students	4,156 (82.2)	4,148 (83.3)	4,623 (84.7)	4, 531 (85.1)
Illicit drug use in the past 12 months†	81 (1.6)	70 (1.4)	111 (2.0)	75 (1.4)
HIV knowledge (mean, 95%CI)‡	6.4 (6.3, 6.4)	6.9 (6.8, 6.9)	7.2 (7.1, 7.3)	7.2 (7.2, 7.3)
Any STI symptoms and signs in the past 12 months#	904 (18.0)	616 (12.4)	740 (13.6)	498 (9.4)

**Note:** *P*<0.001 for all comparisons except for comparison of ethnicity across four study years (*P* = 0.30).

IQR: interquartile range; STI: sexually transmitted infection; CI: confidence interval;

* Kruskal-Wallis equality-of-population rank tests;

** Having a *Hukou* (or residence permit) in the surveyed city;

† Not including alcohol use;

‡ Knowledge score range from 0 to 8.

# Including painful urination, cloudy or bloody discharge from the penis, and ulcers and warts on the penis and around anus, etc.

The strongest predictor for self-reported receipt of any intervention was later survey year. Other respondent characteristics associated with intervention receipt included younger age, single or divorced, higher education, local resident, non-student, study recruitment at sauna or park, younger age of first sex, and any drug use in the past 6 months (Table 3).

**Table 3:** Logistic regression of receipt of any self-reported interventions‡ among MSM in 16 Chinese cities (surveys from all 4 years, 2006–2009).

Variable	*N*	Adjusted OR (95% CI)	*P-value*
Age, every 5-year increase	17709	0.97 (0.94, 0.99)	0.011
Marital status			<0.001
Married or cohabitating	4000	1	
Single or divorced	13707	1.25 (1.14, 1.37)	
Ethnicity			0.036
Han	16291	1	
Other	1418	1.16 (1.01, 1.31)	
Education			<0.001
Middle school or lower	4512	1	
High school	6991	1.20 (1.09, 1.31)	
Some college or higher	6206	1.10 (1.00, 1.22)	
Local residence			<0.001
No	5417	1	
Yes	12292	1.17 (1.08, 1.27)	
Occupation			<0.001
Non-students	14841	1	
Students	2850	0.77 (0.69, 0.85)	
Recruitment venue			<0.001
Bars, Internet or other.	12008	1	
Saunas or parks	5487	1.25 (1.15, 1.35)	
Age of first sex with a man, every 1- year increase	17530	0.98 (0.97, 0.99)	<0.001
Drug use in the past 6 months**			<0.001
No	17400	1	
Yes	294	1.78 (1.27, 2.51)	
Intervention effect by year‡			<0.001
2006	5076	1	
2007	4981	1.81 (1.74, 1.87)	
2008	5460	3.27 (3.04, 3.51)	
2009	5326	5.90 (5.31, 6.57)	

**Note:** CI: confidence interval.

** Not including alcohol use.

‡ These 6 interventions included provision of free condoms, provision of free lubricants, peer education, AIDS/STI publicity materials (pamphlets, brochure, booklet, or foldout), HIV testing or counseling, and testing or treatment of other STIs.

### Condom use rate

There were steadily increasing trends of self-reported condom use over the project years and we could reject the null hypothesis 1. The condom use rate at last sex with a male sexual partner increased from 58% (95% confidence interval [CI]: 57%–60%) in 2006 to 81% (95% CI: 80%–82%) in 2009 (trend test, *P*<0.001), and the consistent condom use rate in the past 6 months increased from 28% (95% CI: 27%–30%) in 2006 to 49% (95% CI: 48%–51%) in 2009 (*P*<0.001) (Table 4).

**Table 4:** Trends of condom use and disease rates among MSM surveyed in 16 Chinese cities from 2006 to 2009.

Variable	2006 (%, 95% CI)	2007 (%, 95% CI)	2008 (%, 95% CI)	2009 (%, 95% CI)	*P*-value*
Condom use at last sex	58.1 (56.6, 59.7)	66.2 (64.7, 67.6)	76.7 (75.4, 77.9)	80.6 (79.5, 81.7)	<0.001
Consistent condom use in past 6 months#	28.3 (26.9, 29.7)	35.7 (34.3, 37.2)	44.5 (43.1, 45.9)	49.2 (47.7, 50.6)	<0.001
HIV prevalence	2.3 (1.9, 2.7)	3.4 (2.9, 4.0)	4.9 (4.3, 5.5)	5.3 (4.7, 5.9)	<0.001
Syphilis prevalence	10.2 (9.3, 11.0)	10.8 (9.9, 11.7)	13.7 (12.8, 14.7)	13.0 (12.1, 13.9)	<0.001

* Cochrane-Armitage trend test;

# 6-month consistent condom use was defined as reported use of condoms during every anal sex episode with a male sexual partner in the past 6 months.

Around 23.9% of participants reported having sex with a female sexual partner, specifically 25.7% in 2006, 20.5% in 2007, 25.9% in 2008 and 23.2% in 2009. The condom use rates at last sex with female sexual partners were 38%, 41%, 49% and 52%, respectively, (not shown in tables); which were lower than the rate with a male sexual partner.

**Figure 2:**
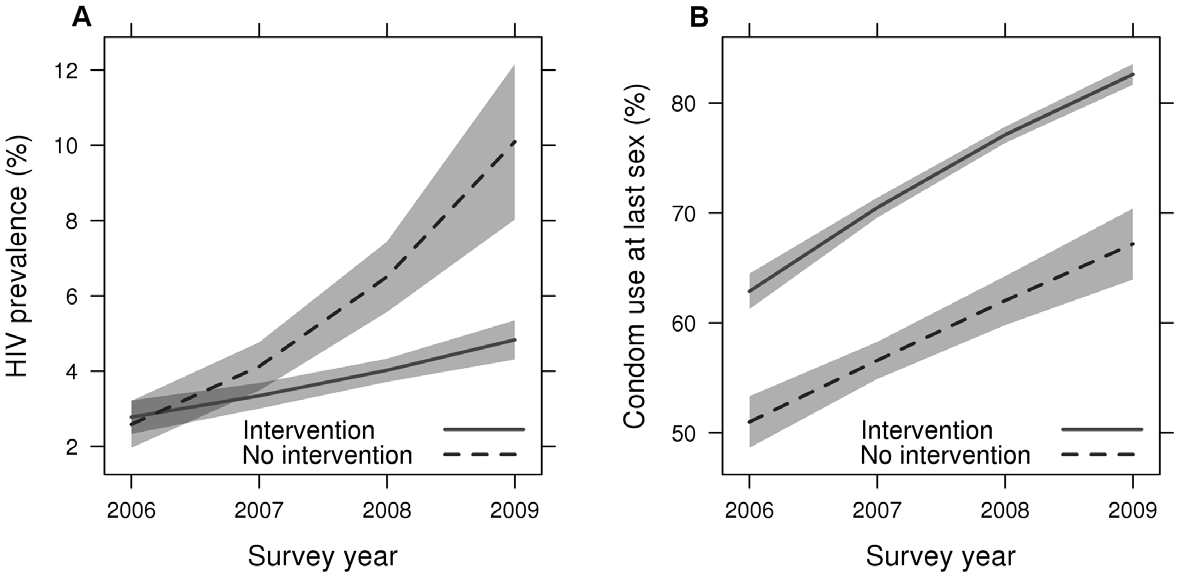
Marginal effects of intervention on condom use at last sex and HIV prevalence among Chinese MSM during 2006–2009 (shadows represent 95% confidence bands): **Figure 2A**: impact on HIV prevalence; **Figure 2B**: impact on condom use.

Over the four project years, the coverage rate of interventions among surveyed MSM samples increased from 59% to 90%, while the average intervention score in all study years was only about half of the maximal value (3.1 out of 6) (Table 5). The self-reported condom use rates at last sex increased in both participants who received interventions and who did not receive interventions in the past 12 months (Table 5, Figure 2B) independent of socio-demographic characteristics, and sex and drug behaviors. There was evidence of an interaction between year and receipt of intervention suggesting that the interventions had a positive effect on condom use over time (*P* = 0.002). Condom use was considerably higher among those who received interventions than those who did not (e.g., pooled 4-year proportion was 55% versus 75%; *P*<0.001).

**Table 5:** Comparison of condom use at last sex and HIV prevalence by receipt of any interventions in the past 12 months among MSM surveyed in 16 Chinese cities from 2006 to 2009.

	2006 (n = 5076)	2007 (n = 4981)	2008 (n = 5460)	2009 (n = 5326)	Pooled (n = 20843)
**Condom use at last sex (%)**					
Did not receive interventions	50.2	53.2	62.3	68.4	55.4
Received interventions	63.8	69.1	79.1	81.9	75.0
Adjusted *P*-value*	<0.001	<0.001	<0.001	<0.001	<0.002***
**HIV prevalence (%)**					
Did not receive interventions	2.1	5.0	6.3	10.3	4.5
Received interventions	2.4	3.1	4.6	4.7	3.9
Adjusted *P*-value*	0.58	0.005	0.057	<0.001	<0.001***
**Coverage of interventions** **	59%	81%	84%	90%	79%
**Intervention score (mean)** #	1.7	3.3	3.6	3.8	3.1

* Adjusted for age, marriage, education, ethnicity, residence, occupation, recruitment venue, age at first sex, and 6-month drug use.

** Coverage rate presents the percentage of participants in each study year who stated they received any of six interventions from the China GF-5 Project in the past 12 months, e.g., 59% said they received interventions in 2006 while 41% did not.

*** There is strong evidence that the association between receipt of interventions and condom use at last sex is different over time (interaction, p = 0.002). There is strong evidence that the association between receipt of interventions and HIV prevalence is different over time (interaction, p<0.001).

# Intervention score ranged from 0–6, and the larger score presents receipt of more interventions in the past 12 months.

### HIV and syphilis prevalence

We also rejected null hypothesis 2, as HIV prevalence increased from 2.3% (95% CI: 1.9%–2.7%) in 2006 to 5.3% (95% CI: 4.7%–5.9%) in 2009 (*P*<0.001) (Table 4). Chongqing City had far higher prevalence than the other 15 cities, but the increasing trends were consistent in all intervention cities (e.g., from 10.1% in 2006 to 19.2% in 2009 in Chongqing City; and from 0.5% in 2006 to 3.5% in 2009 in the other 15 cities) (detailed data not shown). Syphilis prevalence also increased from 10% (95% CI: 9%–11%) in 2006 to 13% (95% CI: 12%–14%) in 2009 (*P*<0.001) (Table 4).

There was evidence of an interaction between year and receipt of interventions suggesting that the interventions had an effect on HIV prevalence over time (*P*<0.001). HIV prevalence increased in both participants who received interventions and who did not receive interventions in the past 12 months; but it increased much more rapidly among participants who did not receive interventions (from 2.1% in 2006 to 10.3% in 2009) than that among those who received interventions (from 2.4% in 2006 to 4.7% in 2009; *P*<0.001) (Table 5, Figure 2A).

### Factors associated with condom use

Older age (adjusted odds ratio [aOR], 0.90; 95% CI, 0.88–0.93) and student status (aOR, 0.76; 95% CI, 0.68–0.84) were associated with lower odds of condom use. Single or divorced marital status (aOR, 1.10; 95% CI, 1.00–1.21) and older age at first sex (aOR, 1.01; 95% CI, 1.00–1.02) had relationship with a higher odds of condom use. Receipt of any intervention in the past 12 months was associated with a higher odds of condom use during every survey year (aOR range, 1.63 to 2.33, *P*<0.001) (Table 6).

**Table 6:** Logistic regression of condom use at last sex with a male partner among MSM in 16 Chinese cities (surveys from all 4 years, 2006–2009).

Variable	*N*	Condom use rate, %	Crude OR (95% CI)	*P-value*	Adjusted OR (95% CI)	*P-value*
Age, every 5-year increase	17709	71.0	0.93 (0.91, 0.94)	<0.001	0.90 (0.88, 0.93)	<0.001
Marital status				<0.001		0.046
Married or cohabitating	4000	5.9	1		1	
Single or divorced	13707	72.5	1.36 (1.26, 1.47)		1.10 (1.00, 1.21)	
Ethnicity				0.49		0.29
Han	16291	71.1	1		1	
Other	1418	70.2	0.96 (0.85, 1.08)		0.93 (0.82, 1.06)	
Education				0.24		0.22
Middle school or lower	4512	70.8	1		1	
High school	6991	71.7	1.05 (0.96, 1.14)		1.07 (0.98, 1.17)	
Some college or higher	6206	70.4	0.98 (0.90, 1.07)		1.07 (0.97, 1.18)	
Local residence				0.07		0.50
No	5417	72.0	1		1	
Yes	12292	70.6	0.94 (0.87, 1.00)		1.03 (0.95, 1.11)	
Occupation				0.003		<0.01
Non-students	14841	71.5	1		1	
Students	2850	68.7	0.88 (0.80, 0.96)		0.76 (0.68, 0.84)	
Recruitment venue				0.92		0.67
Bars, Internet or other.	12008	71.4	1		1	
Saunas or parks	5487	71.3	1.00 (0.93, 1.07)		1.02 (0.94, 1.10)	
Age of first sex with a man, every 1- year increase	17530	71.0	1.02 (0.98, 1.08)	0.33	1.01 (1.00, 1.02)	0.037
Drug use in the past 6 months**				0.98		0.345
No	17400	71.0	1		1	
Yes	294	71.1	1.00 (0.78, 1.29)		0.88 (0.68, 1.14)	
Intervention effect by year‡				<0.001		<0.001
2006	5076	58.1	1.76(1.57, 1.97)		1.63 (1.45, 1.83)	
2007	4981	66.2	1.92 (1.77, 2.08)		1.84 (1.69, 2.00)	
2008	5460	76.7	2.10 (1.89, 2.32)		2.07 (1.87, 2.30)	
2009	5326	80.6	2.29(1.95, 2.68)		2.33 (1.99, 2.74)	

**Note:** CI: confidence interval; STI: sexually transmitted infection.

** Not including alcohol use.

‡ These 6 interventions included provision of free condoms, provision of free lubricants, peer education, STI testing and treatment, HIV testing and counseling, and distribution of AIDS/STI publicity materials (pamphlets, brochure, booklet, or foldout). See also: figure 2b.

### Factors associated with HIV prevalence

College education (aOR, 0.81; 95% CI, 0.67–0.99), local residence in the study cities (aOR, 0.56; 95% CI, 0.48–0.65), student status (aOR, 0.69; 95% CI, 0.53–0.90), and non-Han ethnicity (aOR, 0.60; 95% CI, 0.43–0.82) were associated with lower odds of HIV prevalence rate. Older age (aOR, 1.21; 95% CI, 1.16–1.26), and single or divorced marital status (aOR, 1.37; 95% CI, 1.14–1.65) were associated with a higher odds of HIV prevalence. Receipt of any intervention in the past 12 months was associated with a lower odds of HIV prevalence during every survey year except 2006 (aOR range, 1.09 to 0.45, *P*<0.001) (Table 7).

**Table 7:** Logistic regression of HIV prevalence among MSM in 16 Chinese cities (surveys from all 4 years, 2006–2009).

Variable	*N*	HIV prevalence, %	Crude OR (95% CI)	*P*-value	Adjusted OR (95% CI)	*P*-value
Age, every 5 years increase	20843	4.0	1.15 (1.12, 1.19)	<0.001	1.21 (1.16, 1.26)	<0.001
Marital status				0.465		0.001
Married or cohabitating	4934	4.2	1.00		1.00	
Single or divorced	15907	3.9	0.94 (0.80, 1.11)		1.37 (1.14, 1.65)	
Ethnicity				0.002		0.001
Han	19164	4.1	1.00		1.00	
Other	1679	2.6	0.61 (0.45, 0.83)		0.60 (0.43, 0.82)	
Education				<0.001		0.11
Middle school or lower	5355	5.0	1.00		1.00	
High school	8166	4.0	0.79 (0.67, 0.93)		0.92 (0.77, 1.09)	
Some college or higher	7322	3.2	0.63 (0.53, 0.76)		0.81 (0.67, 0.99)	
Local residence				<0.001		<0.001
No	6125	5.6	1.00		1.00	
Yes	14718	3.3	0.58 (0.51, 0.67)		0.56 (0.48, 0.65)	
Occupation				<0.001		0.007
Non-students	17458	4.3	1.00		1.00	
Students	3363	2.3	0.51 (0.40, 0.65)		0.69 (0.53, 0.90)	
Recruitment venue				0.04		0.57
Bars, Internet or other	14165	3.8	1.00		1.00	
Saunas or parks	6444	4.5	1.17 (1.01, 1.35)		0.96 (0.82, 1.12)	
Age of first sex, every 1- year increase	20843	4.0	1.14 (1.03, 1.26)	0.01	0.98 (0.96, 1.01)	0.15
Drug use in the past 6 months**				0.002		0.001
No	20471	4.0	1.00		1.00	
Yes	337	7.4	1.95 (1.29, 2.95)		2.07 (1.35, 3.18)	
Intervention effect by year‡				<0.001		<0.001
2006	5076	2.3	1.09 (0.81, 1.45)		1.08 (0.80, 1.45)	
2007	4981	3.4	0.80 (0.67, 0.97)		0.80 (0.66, 0.98)	
2008	5460	4.9	0.60 (0.50, 0.71)		0.60 (0.50, 0.71)	
2009	5326	5.3	0.44 (0.34, 0.57)		0.45 (0.34, 0.58)	

**Note:** CI: confidence interval; STI: sexually transmitted infection.

** Not including alcohol use.

‡ These 6 interventions included provision of free condoms, provision of free lubricants, peer education, STI testing and treatment, HIV testing and counseling, and distribution of AIDS/STI publicity materials (pamphlets, brochure, booklet, or foldout). See also: figure 2b.

## Discussion

This Chinese public health program had positive impacts on both behaviors and disease rate among MSM population. Condom use at last sex with a male sexual partner had increased over time, from 58% in 2006 to 81% in 2009. Condom use correlated positively with receipt of the interventions, though the difference of condom use rates between those who received and who did not receive interventions remained 13%–17% over 4 project years. HIV prevalence rates among the pooled surveyed samples from four annual surveys were lower among those who received interventions than those who did not (3.9% versus 4.5%). Although the rates increased in both groups over time, they increased faster among those who received interventions than those who did not; for example, there was no difference in 2006 between groups (2.4% versus 2.1%), but there was a significant difference in 2009 (4.7% in those received interventions versus 10.3% in those who did not).

However, escalation of the coverage and intensity of these interventions is needed for further increasing condom use and for reversing the rising trend of HIV epidemic. While consistent condom use increased from 28% to 49% during the project years, this is still too low to serve as adequate HIV prevention, which may explain why HIV prevalence continued to increase significantly over the course of the study. Several issues regarding the coverage rate and intensity of interventions are worth mentioning. First, the coverage rate is defined as receipt of any of six intervention components, and it reached 90% by 2009. However, as an indicator of intervention intensity, the mean intervention score is low (3.1 out of 6.0). It was suggested that many MSM had missed some components of the intervention package. Second, the coverage rate among the study participants may not represent the overall coverage rate of the interventions for MSM in the cities targeted. Those who did not participate in the surveys may have a lower rate because those who received interventions might be more likely to be enrolled in the surveys.

MSM participants who did not receive interventions also increased condom use over the project years, possibly due to the diffusion of interventions from those who received interventions to their male sexual partners or close friends who even did not receive interventions. However, we did find a 13% to 17% difference of condom use rates between those who received any interventions and those who did not.

Chongqing City has significantly higher HIV prevalence than the other 15 project cities; Chongqing will need a somewhat different and more intense intervention strategy to confront this emergency. Leaders and populations in low prevalence regions are often less aware of the dangers HIV/AIDS present, and lack of sense of urgency to initiate organized responses to tackle this epidemic; in contrast in high prevalence regions, the social infrastructure (e.g. health and education) and basic social services may be overstretched, such that securing resources is both necessary and challenging to establish comprehensive and intensified measures for prevention and care [23]. As China has become the world's second largest economy, major international programs for HIV/AIDS prevention and care have ended or will end soon, e.g. both GF and Bill and Melinda Gates Foundations projects will end in 2012. It is therefore crucial for the local governments in the 15 cities with nascent epidemics to continue to invest in HIV prevention projects in order to prevent the concentrated epidemic in MSM from becoming a generalized epidemic in the whole population. In Chongqing City [24], the strategy should focus on both preventing new infections and treatment as prevention [25]. The Free China CARE program was originally developed for provide cART and care to drug users and plasma donors [15]. MSM patients may face barriers like homosexuality-associated stigma to get full access to comparable services. Chongqing would do well to explore barriers to MSM testing and care, and seek to overcome them with policy change and action.

Our study has several strengths. As an evaluation of public health program in a real-world situation, the findings from this study are particularly useful for policy making. The study participants were recruited from communities in multiple cities, improving generalizability. The study measured both behavioral and biological outcomes in four annual surveys with a substantial sample size; the data can assess trends of these outcomes over time as the coverage and intensity of interventions increased.

The study limitations include a lack of HIV incidence data and the lack of a comparison group. Even though we use participants who did not receive interventions to attempt to derive inferences of program impacts, they are from the same study cities as participants who received interventions and might have been affected by interventions due to intervention diffusion. This could lead to a smaller difference of impacts between two groups than if we had been able to study comparable non-intervention cities. The study design was not a prospective cohort study; therefore, we did not assess temporal relationship between the prevention exposure and the behavioral and disease outcomes. In addition, information on condom use was based on self-reporting, and might be subject to social desirability bias; HIV and syphilis infections were objectively assessed. Moreover, MSM are a marginalized population, and the socially inactive subgroup might be less likely to be reached by project interventions and to be recruited into the surveys through a snowball sampling approach; therefore, our study samples might not fully represent the whole population of MSM in the study cities. The intervention coverage rate and the prevalence rates of condom use and diseases might be overestimated, but the overestimation should not affect the general trends of these outcomes over time.

In conclusion, interventions from the China GF-5 Project were associated with increasing condom use and mitigating the rising trend of HIV infection among MSM. Men who received interventions were more likely to use condoms and had lower HIV prevalence than those who did not receive interventions. It is vital for Chinese public health programs to reach persons who have not received intervention services and increase the coverage and intensity of effective MSM interventions in the future.

## References

[1] Centers for Disease Control and Prevention (2011) HIV in the United States. Available: http://www.cdc.gov/hiv/resources/factsheets/PDF/us.pdf. Accessed 2012 Oct.

[2] World Health Organization Regional Office for Europe (2010) HIV/AIDS Surveillance in Europe. Available: http://ecdc.europa.eu/en/publications/Publications/111129_SUR_Annual_HIV_Report.pdf. Accessed 2012 Oct.

[3] Joint United Nations Programme on HIV/AIDS (2010) UNAIDS Report on the Global AIDS Epidemic 2010. Available: http://www.unaids.org/globalreport/documents/20101123_GlobalReport_full_en.pdf. Accessed 2012 Oct.

[4] World Health Organization Regional Office for South-East Asia. (2009) HIV/AIDS in the Southeast Asia region 2009. Available: http://www.searo.who.int/LinkFiles/Publications_HIV_AIDS_Report2009.pdf. Accessed 2012 Oct.

[5] State Council AIDS Working Committee Office, People's Republic of China (2008) A Joint Assessment of HIV/AIDS Prevention, Treatment and Care in China 2007. Available: http://www.chinaids.org.cn/n435777/n443716/appendix/Joint_Assessment_EN.pdf. Accessed 2012 October.

[6] Ministry of Health, People's Republic of China (2011) National report for HIV/AIDS estimation in China in 2011. Available: http://www.moh.gov.cn/publicfiles///business/cmsresources/mohyzs/cmsrsdocument/doc13944.pdf. Accessed 2012 Oct.

[7] Chow EP, Wilson DP, Zhang J, Jing J, Zhang L (2011) Human immunodeficiency virus prevalence is increasing among men who have sex with men in China: findings from a review and meta-analysis. Sex Transm Dis 38: 845–57. 21844741 10.1097/OLQ.0b013e31821a4f43

[8] Li HM, Peng RR, Li J, Yin YP, et al (2011) HIV incidence among men who have sex with men in China: a meta-analysis of published studies. PLoS One 6: e23431. 21887251 10.1371/journal.pone.0023431 PMC3162552

[9] Liu H, Yang H, Li X, Wang N, Liu H, et al (2006) Men who have sex with men and human immunodeficiency virus/sexually transmitted disease control in China. Sex Transm Dis 33: 68–76. 16432476 10.1097/01.olq.0000187266.29927.11

[10] Qian HZ, Vermund SH (2012) Are Low- and Middle-Income Countries Repeating Mistakes Made by High-Income Countries in the Control of HIV for Men who have Sex with Men? J AIDS Clinic Res S4: e001 doi:10.4172/2155-6113.S4-e001. 10.4172/2155-6113.S4-e001 PMC3893757 24455449

[11] Lai L (2009) The improvement and development of Chinese public health policy since Severe Acute Respiratory Syndrome (SARS) [in Chinese]. Legal System and Society 22: 198–9.

[12] Wu Z, Sullivan SG, Wang Y, Rotheram-Borus MJ, Detels R (2007) Evolution of China's response to HIV/AIDS. Lancet 369: 679–90. 17321313 10.1016/S0140-6736(07)60315-8 PMC7137740

[13] Qian HZ, Hao C, Ruan Y, Cassell HM, Chen K, et al (2008) Impact of methadone on drug use and risky sex in China. J Subst Abuse Treat 34: 391–7. 17869049 10.1016/j.jsat.2007.07.002

[14] Liu B, Sullivan SG, Wu Z (2007) An evaluation of needle exchange programmes in China. AIDS 21: S123–8. 18172380 10.1097/01.aids.0000304707.56670.cf

[15] Zhang F, Haberer JE, Wang Y, Zhao Y, Ma Y, et al (2007) The Chinese free antiretroviral treatment program: challenges and responses. AIDS 21: S143–8. 18172383 10.1097/01.aids.0000304710.10036.2b

[16] Zhang F, Dou Z, Ma Y, Zhao Y, Liu Z, et al (2009) Five-year outcomes of the China National Free Antiretroviral Treatment Program. Ann Intern Med 151: 241–51, W-52. 19687491 10.7326/0003-4819-151-4-200908180-00006

[17] Dou Z, Chen RY, Wang Z, Ji G, Peng G, et al (2010) HIV-infected former plasma donors in rural Central China: from infection to survival outcomes, 1985–2008. PLoS One 5: e13737. 21060835 10.1371/journal.pone.0013737 PMC2966407

[18] Rogowska-Szadkowska D (2011) Consequences of the commercialisation of plasma and blood in China. Przegl Epidemiol 65: 515–9. 22184958

[19] Magnani R, Sabin K, Saidel T, Heckathorn D (2005) Review of sampling hard-to-reach and hidden populations for HIV surveillance. AIDS 19: S67–72. 10.1097/01.aids.0000172879.20628.e1 15930843

[20] Malekinejad M, Johnston LG, Kendall C, Kerr LR, Rifkin MR, et al (2008) Using respondent-driven sampling methodology for HIV biological and behavioral surveillance in international settings: a systematic review. AIDS Behav 12: S105–30. 18561018 10.1007/s10461-008-9421-1

[21] Xiao Y, Sun J, Li C, Lu F, Allen KL, et al (2010) Prevalence and correlates of HIV and syphilis infections among men who have sex with men in seven provinces in China with historically low HIV prevalence. J Acquir Immune Defic Syndr 53: S66–73. 20104113 10.1097/QAI.0b013e3181c7db43

[22] Xiao Y, Ding X, Li C, Liu J, Sun J, et al (2009) Prevalence and correlates of HIV and syphilis infections among men who have sex with men in Chongqing Municipality, China. Sex Transm Dis 36: 647–56. 19955876 10.1097/OLQ.0b013e3181aac23d

[23] United Nations Population Fund (2012) HIV Prevention Now - Programme Briefs. Available: http://www.unfpa.org/hiv/prevention/index.htm. Accessed 2012 October.

[24] Feng L, Ding X, Lu R, Liu J, Sy A, et al (2009) High HIV prevalence detected in 2006 and 2007 among men who have sex with men in China's largest municipality: an alarming epidemic in Chongqing, China. J Acquir Immune Defic Syndr 52: 79–85. 19448559 10.1097/QAI.0b013e3181a4f53e

[25] Cohen MS, Chen YQ, McCauley M, Gamble T, Hosseinipour MC, et al (2011) Prevention of HIV-1 infection with early antiretroviral therapy. N Engl J Med 365: 493–505. 21767103 10.1056/NEJMoa1105243 PMC3200068

